# Retraction Note: Tumor-specific Th2 responses inhibit growth of CT26 colon-cancer cells in mice via converting intratumor regulatory T cells to Th9 cells

**DOI:** 10.1038/s41598-021-92334-5

**Published:** 2021-06-16

**Authors:** Jiang-Qi Liu, Xing-Yong Li, Hai-Qiong Yu, Gui Yang, Zhi-Qiang Liu, Xiao-Rui Geng, Shuai Wang, Li-Hua Mo, Lu Zeng, Miao Zhao, Yun-Ting Fu, Hong-Zhi Sun, Zhi-Gang Liu, Ping-Chang Yang

**Affiliations:** 1 ENT Institute of Shenzhen University School of Medicine and Shenzhen Key Laboratory of Allergy and Immunology, Shenzhen, 518060 China; 2 Department of Surgery, The First Hospital, Liaoning Medical University, Jinzhou, 100044 China; 3 Longgang Central Hospital, Shenzhen, 518116 China; 4 Brain Body Institute, McMaster University, Hamilton, ON L8N 4A6 Canada

Retraction of: *Scientific Reports* 10.1038/srep10665, published online 02 June 2015

The Editors have retracted this Article.

After publication, concerns were raised in regard to partial overlap of the flow cytometry data presented in Figure 3. In addition, the authors were not able to locate the original data files. The Editors therefore no longer have confidence in the integrity of the data presented.

The authors do not agree with the retraction of this Article.

